# Supplementary figures and images for: Behavior test for seven-week old puppies (Canis familiaris): Inter-rater reliability and factors associated with test performance
Source: PLoS One. 2020 Jul 29;15(7):e0236271. doi: 10.1371/journal.pone.0236271 (PMC7390333; doi:10.1371/journal.pone.0236271)

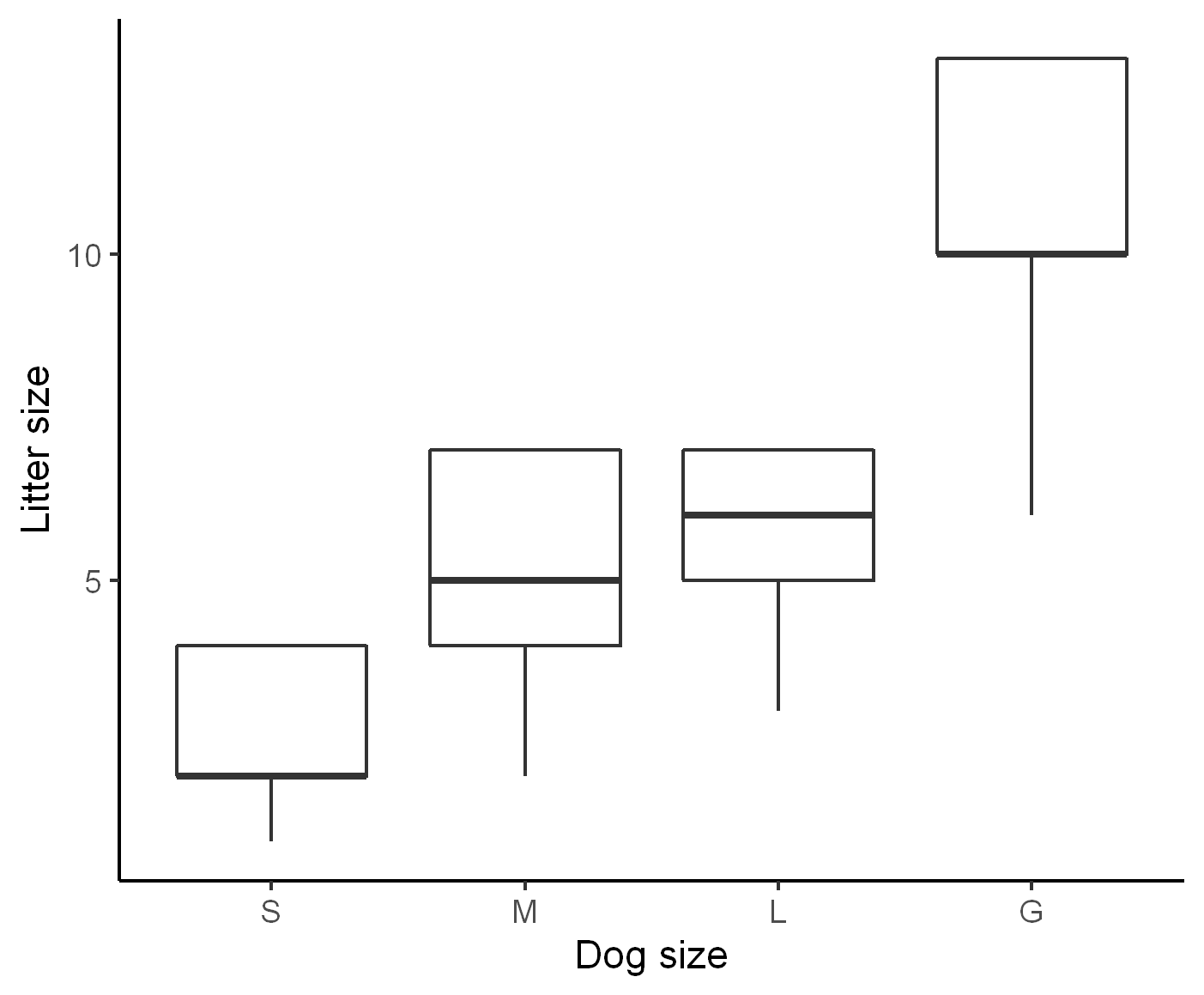

Supplement: S1 Fig — (PNG) [file pone.0236271.s002.png]
